# A triage framework for managing novel, hybrid, and designed marine ecosystems

**DOI:** 10.1111/gcb.14757

**Published:** 2019-08-13

**Authors:** Marie‐Lise Schläppy, Richard J. Hobbs

**Affiliations:** ^1^ Faculty of Engineering and Mathematical Sciences Oceans Graduate School The University of Western Australia Crawley WA Australia; ^2^ Australian Institute of Marine Science IOMRC (M096) Crawley WA Australia; ^3^ School of Biological Sciences The University of Western Australia Crawley WA Australia

**Keywords:** climate change, conservation, human values, marine ecosystem, marine phase shifts, novel ecosystem, restoration

## Abstract

The novel ecosystem (NE) concept has been discussed in terrestrial restoration ecology over the last 15 years but has not yet found much traction in the marine context. Against a background of unprecedented environmental change, managers of natural marine resources have portfolios full of altered systems for which restoration to a previous historical baseline may be impractical for ecological, social, or financial reasons. In these cases, the NE concept is useful for weighing options and emphasizes the risk of doing nothing by forcing questions regarding the value of novelty and how it can best be managed in the marine realm. Here, we explore how the concept fits marine ecosystems. We propose a scheme regarding how the NE concept could be used as a triage framework for use in marine environments within the context of a decision framework that explicitly considers changed ecosystems and whether restoration is the best or only option. We propose a conceptual diagram to show where marine NEs fit in the continuum of unaltered to shifted marine ecosystems. Overall, we suggest that the NE concept is of interest to marine ecologists and resource managers because it introduces a new vocabulary for considering marine systems that have been changed through human actions but have not shifted to an alternate stable state. Although it remains to be seen whether the concept of marine NEs leads to better conservation and restoration decisions, we posit that the concept may help inform management decisions in an era of unprecedented global marine change.

## Graphical abstract

The novel ecosystem (NE) concept has been discussed in terrestrial restoration ecology over the last 15 years but has not yet found much traction in the marine context. Here the NE concept is explored for marine ecosystems and a triage framework is proposed to specifically consider ecosystems that have been altered by human impacts but have not yet shifted to an alternative stable state.

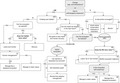

## 1 INTRODUCTION

Managers of marine ecosystems are faced with making decisions about how to manage systems in various states of alteration. There is an imperative to ensure that often‐scarce conservation funds are spent wisely. Here we explore the potential of a triage framework that builds on, and expands, the concept of novel ecosystems (NEs) in a marine context. The NE concept has arisen in the field of terrestrial ecology because humans are driving many abiotic and biotic changes on the planet. The link between human development and the depletion of natural resources or modification of the environment is so evident that the term “Anthropocene” has been used to describe this era (Steffen, Grinevald, Crutzen, & McNeill, 2011). With the number of impacted marine ecosystems increasing, difficult conservation and restoration decisions need to be made against a background of finite resources to fund their restoration. The pace of change is accelerating (Steffen, Broadgate, Deutsch, Gaffney, & Ludwig, 2015), and the prioritization of restoration efforts may be increasingly needed.

The NE concept was coined by Chapin and Starfield (1997). The working definition of NE has evolved rapidly over the last 15 years, starting with a purely biological focus (see Hobbs et al., 2006) and shifting to the current definition, which also encompasses a social dimension, grounding the concept firmly within the idea that all ecosystems are, in fact, socioecological systems (Backstrom, Garrard, Hobbs, & Bekessy, 2018):

A novel ecosystem is a system of abiotic, biotic and social components (and their interactions) that, by virtue of human influence, differ from those that prevailed historically, having a tendency to self‐organise and manifest novel qualities without intensive human management. Novel ecosystems are distinguished from hybrid ecosystems by practical limitations (a combination of ecological, environmental and social thresholds) on the recovery of historical qualities.(Hobbs, Higgs, & Hall, 2013, p. 58)

A terrestrial example of an NE is the Mt. Sutro forest in San Francisco, which is composed of almost entirely non‐native species and is dominated by Australian eucalyptus (Venton, 2013). Historically, the area is assumed to have been dominated by chaparral shrubland, although there is little concrete documentation. The forest was planted with eucalyptus in the late 19th century. This system now functions as a fog forest, trapping coastal fog regularly and maintaining mesic conditions. The authority in charge of the area developed plans to remove most of the eucalyptus and return native vegetation to some or all of the site on the basis that eucalyptus constituted a fire hazard (Venton, 2013). However, the current forest composition is arguably less of a fire hazard than the putative native vegetation because of its fog‐trapping capacity. In addition, the forest is greatly valued by the local community. There are thus strong social barriers to the removal of the existing system and the recovery of historical qualities, especially given the significant doubt about what these historical qualities actually were.

Thus, Mt. Sutro illustrates all the prime qualities of an NE: a biological assemblage that is self‐organizing and functional but different from what would have been in place previously that poses important social and ecological questions regarding traditional restoration or conservation options. Some commentators have argued that NEs are simply degraded systems in need of restoration (Murcia et al., 2014). However, how degradation is defined and perceived is value‐laden and context‐dependent, and simply calling an altered system “degraded” may ignore important values that the altered system retains or develops (Hobbs, 2016).

The motivation behind developing the NE concept derives from the need to prioritize conservation and restoration actions in the face of ongoing inadequate budgets and resources that are allocated to these tasks. The aim is to focus traditional restoration and conservation activities in areas where they will be most effective while considering alternative strategies for altered systems that may have important values as they exist now (Hobbs, Higgs, & Hall, 2017; Hobbs et al., 2014). In this regard, the concepts of novel and hybrid ecosystems fit into the current trend in using ecological triage to help managers making difficult decisions about altered ecosystems (Hobbs & Kristjanson, 2003; Rappaport, Tambosi, & Metzger, 2015).

The key components of the NE definition are that the drivers of novelty are anthropogenic, that the ecosystems have been brought about by biotic and abiotic changes, and that the novel assemblages and interactions persist without human intervention (Higgs, 2017; Hobbs et al., 2013). A key element of the early definitions of NEs was that there were ecological, practical, or social thresholds in place that prevented effective restoration back to a historical baseline. Where such thresholds were not present, there was potential to restore the system. These systems were called “hybrid” systems, indicating that they had undergone change but retained the potential for restoration. The various elements of this definition continue to be debated and modified by various commentators, with some suggesting that the distinction between hybrid and novel systems is not useful because relevant thresholds are often difficult to recognize (e.g., Miller & Bestelmeyer, 2016). Still, it may be useful in management contexts to establish a categorical difference between systems that are candidates for restoration and those that are unlikely to be restored.

According to the principles behind the NE concept (Hobbs et al., 2006, 2013, 2014), ecosystems that are not selected for restoration may still have inherent value even if these systems no longer follow the ecological trajectory of the historical system. If the value of these systems can be measured and is found to be too low, then their trajectory could be corrected to produce more desirable outcomes.

To date, most discussions of NEs have focused on terrestrial or freshwater systems, and relatively little attention has been paid to novel marine systems (but see Graham, Cinner, Norström, & Nyström, 2014; Harborne & Mumby, 2011; Perring & Ellis, 2013). Here, we discuss the NE concept within the context of marine systems. We explore whether NEs can be used as a triage framework to prioritize marine conservation actions and how the NE concept fits with commonly used ecosystem state descriptors in marine science (i.e., phase shifts).

## 2 NOVEL MARINE ECOSYSTEMS

The NE concept may be of value to marine ecologists, biologists, and marine conservation managers because, just like their terrestrial counterparts, marine ecosystems are faced with rapid environmental change, limited global political will to address these changes, and the lack of resources to remedy the changes.

Traditional conservation and environmental management methods for marine ecosystems and resources have included marine protected areas, catch size limits, quotas, and temporary closures for fisheries. The ability of some of these measures to foster the resilience of some marine ecosystems (e.g., coral reefs) is being increasingly questioned (e.g., Bruno, Côté, & Toth, 2018). Many marine scientists anticipate that the speed of climate change will result in the loss of marine ecosystem resilience, biodiversity, and structural complexity, and reduce the survivability of calcifying organisms (Fabricius et al., 2011; Orr et al., 2005). When standard marine conservation measures have limited success or fail altogether, marine ecologists and managers increasingly recognize that restoration needs to be attempted (Anthony et al., 2017), although the underlying conditions causing the perturbation of these marine ecosystems are likely to endure into the future. In addition, there is growing recognition of the potential for human‐created habitats in the form of various types of infrastructure (oil platforms, artificial reefs, etc.) to provide important conservation resources.

In marine systems, the difficulties (logistical and financial) associated with restoration are substantial. Conservative estimates by Bayraktarov et al. (2015) showed that the cost of restoring marine coastal habitat was 80,000 USD per hectare (median), which may be the reason why marine managers have prioritized conservation initiatives over restoration to date. The amount of time, effort, and funds dedicated to the restoration of one ecosystem may restrict the resources available for other conservation activities, and therefore, tools that aid in the prioritization of conservation and restoration targets are needed because the consequences of delaying action may also amount to making a decision.

**Figure 1:**
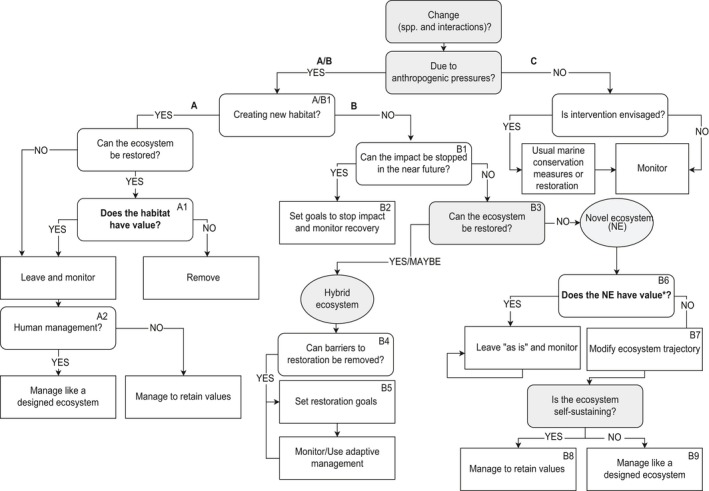
A marine ecosystem decision‐making framework setting out decisions relating to different types of alteration. A: addition of marine habitat through man‐made structures. B: application of novel ecosystems concepts as derived from terrestrial systems (Hobbs et al., 2014). Additional social dimensions relating to values are indicated in bold text. C: marine ecosystem management in a “business‐as‐usual” scenario using commonly used marine conservation measures. *Values refer to the end‐state values defined in Wallace et al. (2018)

In this sense, the NE concept aligns with the triage concept of prioritizing restoration or conservation action at sites with the greatest need or threat or those that would receive the greatest benefit or have the best chance for recovery (Bottrill et al., 2008, 2009; Hobbs & Kristjanson, 2003). Other decision‐making methods are also available, such as the Investment Framework for Environmental Resources (http://www.inffer.org), the project prioritization protocol (Joseph, Maloney, & Possingham, 2009), or Bayesian logic (Stewart et al., 2013), but the NE concept may become another tool for conservation prioritization. The NE concept and triage are not universally adopted because these concepts can lead to the passive acceptance of the lack of resources assigned to conservation, which may foster negative conservation outcomes, such as species extinction (Jachowski & Kesler, 2009; Parr et al., 2009) and touches on values associated with conservation triage (Wilson & Law, 2016). In this sense, the well‐articulated and valid points against the NE concept by Murcia et al. (2014) and Aronson et al. (2014) mirror concerns expressed against triage in environmental management. For those who find the idea of triage acceptable, the NE concept can help clarify which ecosystems need their value assessed or their trajectory modified (by using the decision tree in Figure 1). The scheme integrates components of the NE diagram presented in Hobbs et al. (2014) (Figure 1, B, gray elements) but also includes other components related to the value of ecosystems on human well‐being (Figure 1, A and B in bold) and the often‐encountered situation in marine systems where man‐made structures are placed in oceans (Figure 1, Section A). Although the material in Section A is also potentially relevant in the terrestrial realm, the content is currently of particular interest in the marine realm and the topic of much debate, and hence, the material is included here.

### 2.1 Marine habitat creation

Many man‐made structures are placed in marine environments: jetties, harbors, breakwaters, wrecks, navigational aids, and energy‐producing infrastructure (oil and gas, offshore wind turbines, waves and tidal devices). These structures create new hard substrata for marine species and often create a vertical point of reference for marine organisms to recruit. Many species that colonize this kind of hard substrata are seen as desirable (corals, sponges, mussels, and other sessile invertebrates) and attract associated species of mobile macroinvertebrates and fish, leading to an increase in biodiversity (Langhamer, Wilhelmsson, & Engström, 2009; van der Stap, Coolen, & Lindeboom, 2016; Wilhelmsson et al., 2010). The less desirable effects associated with the addition of man‐made structures in marine environments are the risks of creating stepping stones for non‐native and invasive species (Creed et al., 2017) and the contribution to ocean sprawl (Heery et al., 2017). Currently, the value of these structures is particularly being scrutinized, as many oil and gas fields are coming to the end of their production lives and will soon be in need of decommissioning. The ecological value of these structures and whether in situ decommissioning is likely to foster the survival of natural communities will be a question of increasing importance in the next 10–20 years (Cullinane & Gourvenec, 2017; NERA, 2016).

Non‐ecological values will also need to be taken into consideration because the importance of human values in decision‐making concerning natural resources is widely acknowledged (Heink & Jax, 2019; Keeney, 2006; Kenter et al., 2015; Shields, 2002). Ideally, the values derived from marine man‐made structures would be assessed as part of the process of deciding their fates (remove or retain) (Figure 1, A1). For this, a rapid and efficient method is needed, and rather than estimating the ecosystem services values provided by the structures, we endorse the method of value elicitation proposed by Wallace, Wagner, and Smith (2016). The concepts behind ecosystem services are still being debated actively in the literature, and the link to human well‐being seems to be a promising avenue for determining the value of natural assets (Heink & Jax, 2019). Therefore, the method of Wallace et al. (2016) is highly applicable given that it is specifically designed for group deliberative processes and expert knowledge elicitation when decisions have to be made and limited resources are available. As Backstrom et al. (2018) argue, the NE concept is grounded in social systems, and when used as a decision‐making tool, the concept does not solely exist in the biological sciences. Therefore, the full range of values that may be derived from NEs should be assessed to inform environmental management. The human values proposed represent “desirable end‐states of existence for humans” (Wallace et al., 2016, p. 162). “End‐state” values are “enduring beliefs concerning the preferred end‐state of human existence, including those required for survival and reproductive success, which taken together determine human wellbeing” (Wallace, Kiatkoski, Rogers, & Jago, 2018, p. 5); these values represent the penultimate answer to the question, “why is this important?” These “end‐state” values are thus structured in a form that supports the analysis of trade‐offs, synergies, and overall utilities of different environmental management options (Wallace & Jago, 2017; Wallace et al., 2018). Whether an NE contributes to human well‐being through one of the “end‐state” values has to be effectively assessed to compare options.

If leaving man‐made structures in the ocean is of high value to key stakeholders, then the next decision concerns how much management the structures require to sustain various biological assemblages (Figure 1, A2). If an ecosystem needs regular management to maintain its ecological trajectory and the value it provides, the ecosystem is designed rather than novel (Higgs, 2017); consequently, the ecosystem should be managed as such rather than as an NE that follows its own trajectory without human intervention (Hobbs et al., 2017).

In the marine context, man‐made structures often have a primary function (and associated values), and their use by marine species gives them a value that is only secondary until the structure is earmarked for removal. These structures may act as artificial reefs, but they were not created with the intention of providing a specific environmental value or service; hence there is a need to measure what their value or service might be. In short, we advise determining the values of these structures to stakeholders before decommissioning decisions are made.

### 2.2 Prioritizing the restoration of marine ecosystems

When the changes observed in a community are not attributable to the addition of man‐made structures but are of anthropogenic origin (Figure 1, B), the question is whether the impact can be stopped at timescales relevant to the natural recovery of the system (Figure 1, B1). If the impact cannot be stopped, the ongoing management of chronic ecosystem damage will be required (Figure 1, B2). If the impact cannot be stopped and the ecosystem is unable to recover naturally, restoration might be needed (Figure 1, B3). The determination as to whether an ecosystem can be brought back to a chosen historical baseline is at the core of the NE concept (Hobbs et al., 2006). Modeling may be the best tool to determine whether an ecosystem can recover naturally, but recovery will only be possible if the rate of ecosystem degradation is slow and if the research needed to parameterize the model has already been carried out or can be undertaken rapidly. In cases where the impact acts quickly on the ecosystem, time will be lacking to obtain the research results needed to underpin the model. In that case, this question may be best answered by expert opinion elicitation, for which the Wallace et al. (2016) method can be used. If all barriers to restoration can be removed (Figure 1, B4), temporal and spatial restoration targets can be specified (Figure 1, B5) while bearing in mind that human impacts are still present. These impacts will continue to affect these marine communities and likely affect the restoration outcomes.

When an altered system cannot be restored to a chosen historical baseline, the value of the novelty needs to be the focus of research (Figure 1, B6). Some altered systems may have inherent value, warranting that they be kept in a manager's portfolio and left to follow their own trajectory. Alternatively, if their value is low, a manager might choose to alter the ecosystem trajectory by intervening and pushing the ecosystem toward a state that is perceived to have a greater value (Figure 1, B7). The value of an NE is context‐dependent and may be different at different geographical locations (see Box 1). If an NE is self‐sustaining after modifying its trajectory (Figure 1, B8), what was seen as the novelty in that ecosystem becomes an inherent characteristic of that ecosystem against which the next round of novelty can be measured. This is essentially a shifting baseline, but in this case, the process is documented. If the new trajectory is dependent on continued human intervention for its positive values, then the ecosystem can be managed similar to a designed ecosystem (Higgs, 2017); (Figure 1, B9).

BOX 1 **Applying the NE concept to a real‐life marine scenario**

1 The Great Barrier Reef of Australia (GBR) has experienced elevated sea surface temperatures over two consecutive summers, and in some sections, over 50% of the reef was bleached 2 years in a row (Hughes et al., 2018). The recovery potential is unknown, and if managers would like to explore restoration options rather than relying on self‐regeneration, the NE concept can be used to inform decisions about what and when to restore. The restoration of the entire GBR is a daunting prospect, and scaling up restoration efforts over approximately 2,000 km may be practically impossible. While broad policy and management changes (for instance, fisheries measures such as banning herbivorous fish catches on reefs) are likely to be needed for effective conservation outcomes, local management and restoration efforts will be required. The decision tree in Figure 1 was used for two hypothetical regions of the GBR: Section 1: a southern section close to Airlie Beach; and Section 2: a far north section far from Cooktown. The relevant boxes from Figure 1 are indicated in brackets in the text.

Starting at the top of Figure 1, changes were observed in the species composition: bleaching has impacted the GBR, leading to the death of a substantial proportion of branching corals. The impact is anthropogenic because bleaching is caused by climate change, the speed of which is exacerbated by human activities (Cook et al., 2016). This then leads to the A/B side of the diagram. Given that no new habitat has been created (A/B1), Section B of the diagram is then followed.

Section 1:

- Can the ecosystem be restored? (B3): restoration may be possible due to the proximity to a township with the political will to restore the reef for tourism, making Section 1 a hybrid.
- The restoration efforts of Section 1 (B4, B5) would need to be applied locally (e.g., branching coral transplantation), and national action to remedy the cause of bleaching (e.g., action on carbon emissions) must be undertaken so that the undelaying threat is removed in the future.

Section 2:

- Can the ecosystem be restored? (B3): no, the logistics of restoration due to the geographical location and scale are too difficult and costly. Modeling should be carried out to estimate the chances of natural recovery. If the model shows no potential for recovery, the coral reef would be novel. This identifies Section 2 as an NE, and its value would have to be measured (B6). End‐state value elicitation (Wallace et al., 2016) for all major stakeholders of Section 1 could be carried out. Does the novel reef still support end‐state values, such as “a benign environment” (i.e., coastal protection) (sensu Wallace et al., 2018, p. 9)? If yes, then the reef can be left as is. If the NE has lost its value, managers could attempt to alter Section 1's trajectory (B7). The opportunities for doing this will be context‐ and resource‐dependent but might include introducing artificial structures that mimic the 3‐D structure of a coral reef and removing or reintroducing key ecosystem‐building species (e.g., promoting an oyster/mussel reef). In a marine park, the options to create a different self‐sustaining ecosystem might be limited, but in another area, the option might be feasible. What the ecosystem might look like will depend on the needs of the stakeholders. The new ecosystem would have to be self‐sustaining (B8) or else it would become a designed ecosystem (B9) that needs constant management to retain its value, which, given the location of Section 2, would not be feasible.

Although the whole GBR has experienced the same impact, the decisions about how to handle the ecosystem that ensues can be different in different locations, and the scheme in Figure 1 may help in asking pertinent questions about ecosystem trajectories.

### 2.3 Usual management tools and strategies

In Figure 1, C, if the changes in species assemblages and interactions are not due to human‐induced drivers and if intervention is envisaged, then the usual marine management tools, such as implementing marine protected areas, setting fisheries quotas, and temporary closures, can be used to help the system recover. Restoration actions, such as restoring a coral reef dive site used by the tourism industry after a tropical cyclone, can also be envisaged. In both cases, intervening or not intervening, marine monitoring is paramount and must be the basis on which future decisions are informed. Long‐term monitoring can help identify ecosystem changes and trajectories that need to be understood to inform management decisions.

## 3 HOW WELL DOES THE NE CONCEPT APPLY TO MARINE ECOSYSTEMS?

From its inception, the NE concept was essentially defined as an ecosystem state in which biological metrics, such as a change in species composition and interactions, could be measured, and compared to a historical baseline (Hobbs et al., 2006). In the marine realm, baselines are lacking for many ecosystems, and there is often limited or no knowledge of historical trajectories. In addition, conservation biology, including marine conservation, is complicated by the shifting baseline issue, where each generation compares the changes they witness to an ecosystem state seen in their lifetimes, when in fact, the system has degraded significantly compared to the baseline used by a previous generation (Pauly, 1995). Therefore, the baseline that restoration is attempting to re‐establish needs to be very clearly defined, and the restored state may never approach the original pristine state (which in itself might be difficult to define). If no baseline is available, the answer to the question “can the ecosystem be restored to a historical baseline?” is “no,” making it an NE by default.

The NE concept cannot practically be applied to all regions of the marine realm because restoration is not feasible in international waters and the open ocean. Nevertheless, novelty in these ecosystems can be discussed in theoretical terms (see Harborne & Mumby, 2011; Perring & Ellis, 2013), but when decisions must be made about the practical feasibility of restoration, the NE concept can be considered only for ecological units that are in the jurisdiction of a marine manager or management body with agency to act.

**Figure 2:**
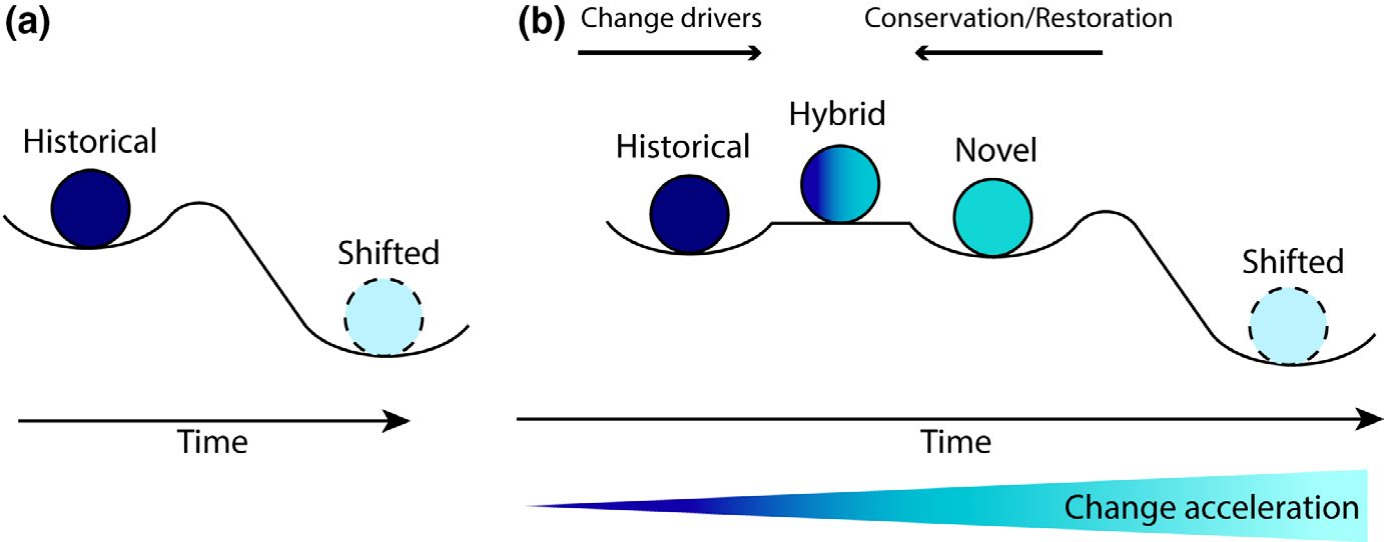
Conceptual diagram proposing a place for novel ecosystems (NEs) in relation to phase‐shifted ecosystems: (a) a phase‐shift scenario where the threshold separating a historical system and a shifted system is biological only and (b) an NE scenario where the historical/hybrid/novel state is separated from the hybrid/novel/shifted state by biological or social thresholds. Note that the troughs are not quantitatively representative. The hybrid state is stable when the drivers of change and the conservation and restoration efforts are equal. The state can be pushed back to a historical state through restoration efforts

Unlike a system that has undergone a phase shift (Figure 2a), a novel marine ecosystem is a system that has retained enough of the species from its former habitat (sensu Jones & Andrew, 1992) but where the species richness or abundance of the ecosystem‐building species has been altered compared to a chosen historical baseline. For example, a coral reef where the species of coral susceptible to bleaching have died (i.e., branching morphologies; Marshall & Baird, 2000) can be considered an NE (Graham et al., 2014) if restoration cannot be attempted. The word “novel,” whose merit (or lack thereof) is being debated in the field of terrestrial ecology, can be used to describe altered marine ecosystems that have not yet undergone a phase shift (Figure 2b). It is probable that all ecosystems on the planet have the potential to turn into hybrid novel or phase‐shifted ecosystems, and the difference between the phases is a function of the resilience of the system and the amount of restoration effort directed toward these systems (Figure 2b). Dudney, Hobbs, Heilmayr, Battles, and Suding (2018) identified the need to more clearly identify the relationships between novelty and resilience, and the authors have recently explored the complex intersection of ecological novelty and resilience‐based management in detail.

## 4 CONCLUSION

The idea of prioritizing areas for restoration and conservation action has not been embraced by all ecologists and managers (Jachowski & Kesler, 2009; Parr et al., 2009), but prioritization may become increasingly acceptable as we witness the demise of a number of ecosystems due to human impacts. Compared to other schemes, the NE concept as a prioritization tool still needs to be evaluated in more detail and tested empirically. The NE concept allows marine ecologists and managers to share a vocabulary for discussing changing marine systems in areas where restoration is unlikely or impossible (e.g., the deep sea, international waters) and systems that have not shifted to an alternate stable state. When envisaging conservation or restoration actions, the decision tree in Figure 1 aids in the identification of where research is needed, whether it be ecological or social.

The consideration of NEs fosters our ability to ask pertinent questions that go beyond the realm of natural sciences and emphasize the fact that in an era of rapid global change, the value of natural systems cannot be considered solely on the basis of their intrinsic biological or ecological value.

Some limitations of the concept arise due to its multiple dimensions (social and biological). Thresholds such as those proposed in alternate stable state theory (Holling, 1973) are notoriously difficult to define (Capon et al., 2015; Seastedt, Hobbs, & Suding, 2008). Determining at what threshold a hybrid ecosystem will turn into an NE is potentially problematic. To date, empirical evidence indicating that using the NE concept yields superior environmental management outcomes is not readily available.

Nevertheless, when presented to ecosystem managers and those engaged in the restoration of these ecosystems, the NE concept seems to make intuitive sense, and the number of scholarly publications on this topic shows that there is interest in the questions the concept raises. In the terrestrial realm, the NE concept has put words to what people were observing and experiencing as they tried to manage or restore systems. The NE concept also adds new tools to the conservation arsenal by recognizing that altered/new systems may have value as they currently exist, allowing scarce management dollars to be spent on other conservation initiatives. Whether the adoption of the NE concept is a useful addition to or a substitute for current triage practices remains to be investigated.

Overall, we find the NE concept to be promising for use in marine ecology and restoration only if the words “novel” and “novelty” are not used to incentivize causing environmental impacts with impunity. The concept can be used to emphasize the real threat of “doing nothing” in the face of global biotic and abiotic changes in marine ecosystems. Further work in this area could include a systematic review of marine ecosystems to assess if and how the concept could be applied in practice and whether the application could lead to more transparent and successful conservation outcomes.

## CONFLICT OF INTEREST

On behalf of all authors, the corresponding author states that there is no conflict of interest.

## Data Availability

No data were used for this study.

## References

[gcb14757-bib-0001] Anthony, K. , Bay, L. K. , Costanza, R. , Firn, J. , Gunn, J. , Harrison, P. , … Walshe, T. (2017). New interventions are needed to save coral reefs. Nature Ecology & Evolution, 1(10), 1420–1422. 10.1038/s41559-017-0313-5 29185526

[gcb14757-bib-0002] Aronson, J. , Murcia, C. , Kattan, G. H. , Moreno‐Mateos, D. , Dixon, K. , & Simberloff, D. (2014). The road to confusion is paved with novel ecosystem labels: a reply to Hobbs et al. Trends in Ecology & Evolution, 29(12), 646–647. 10.1016/j.tree.2014.09.011 25445875

[gcb14757-bib-0003] Backstrom, A. C. , Garrard, G. E. , Hobbs, R. J. , & Bekessy, S. A. (2018). Grappling with the social dimensions of novel ecosystems. Frontiers in Ecology and the Environment, 16(2), 109–117. 10.1002/fee.1769

[gcb14757-bib-0004] Bayraktarov, E. , Saunders, M. I. , Abdullah, S. , Mills, M. , Beher, J. , Possingham, H. P. , … Lovelock, C. E. (2015). The cost and feasibility of marine coastal restoration. Ecological Applications, 26(4), 1055–1074. 10.1890/15-1077.1 27509748

[gcb14757-bib-0005] Bottrill, M. C. , Joseph, L. N. , Carwardine, J. , Bode, M. , Cook, C. , Game, E. T. , … Possingham, H. P. (2008). Is conservation triage just smart decision making? Trends in Ecology & Evolution, 23(12), 649–654. 10.1016/j.tree.2008.07.007 18848367

[gcb14757-bib-0006] Bottrill, M. C. , Joseph, L. N. , Carwardine, J. , Bode, M. , Cook, C. , Game, E. T. , … Possingham, H. P. (2009). Finite conservation funds mean triage is unavoidable. Trends in Ecology & Evolution, 24(4), 183–184. 10.1016/j.tree.2008.11.007

[gcb14757-bib-0007] Bruno, J. F. , Côté, I. M. , & Toth, L. T. (2018). Climate change, coral loss, and the curious case of the parrotfish paradigm: Why don't marine protected areas improve reef resilience? Annual Review of Marine Science, 11(1), 307–334. 10.31230/osf.io/ugk4v 30606097

[gcb14757-bib-0008] Capon, S. J. , Lynch, A. J. J. , Bond, N. , Chessman, B. C. , Davis, J. , Davidson, N. , … Nally, R. M. (2015). Regime shifts, thresholds and multiple stable states in freshwater ecosystems; a critical appraisal of the evidence. Science of the Total Environment, 534, 122–130. 10.1016/j.scitotenv.2015.02.045 25712747

[gcb14757-bib-0009] Chapin, I. F. S. , & Starfield, A. M. (1997). Time lags and novel ecosystems in response to transient climatic change in arctic Alaska. Climatic Change, 35(4), 449–461.

[gcb14757-bib-0010] Cook, J. , Oreskes, N. , Doran, P. T. , Anderegg, W. R. L. , Verheggen, B. , Maibach, E. W. , … Rice, K. (2016). Consensus on consensus: A synthesis of consensus estimates on human‐caused global warming. Environmental Research Letters, 11(4), 048002 10.1088/1748-9326/11/4/048002

[gcb14757-bib-0011] Creed, J. C. , Fenner, D. , Sammarco, P. , Cairns, S. , Capel, K. , Junqueira, A. O. R. , … Oigman‐Pszczol, S. (2017). The invasion of the azooxanthellate coral *Tubastraea* (*Scleractinia*: *Dendrophylliidae*) throughout the world: History, pathways and vectors. Biological Invasions, 19(1), 283–305. 10.1007/s10530-016-1279-y

[gcb14757-bib-0012] Cullinane, B. , & Gourvenec, S. (2017). Decommissioning – The next Australian oil and gas boom? The APPEA Journal, 57(2), 421–425. 10.1071/aj16203

[gcb14757-bib-0013] Dudney, J. , Hobbs, R. J. , Heilmayr, R. , Battles, J. J. , & Suding, K. N. (2018). Navigating novelty and risk in resilience management. Trends in Ecology & Evolution, 33(11), 863–873. 10.1016/j.tree.2018.08.012 30268524

[gcb14757-bib-0014] Fabricius, K. E. , Langdon, C. , Uthicke, S. , Humphrey, C. , Noonan, S. , De'ath, G. , … Lough, J. M. (2011). Losers and winners in coral reefs acclimatized to elevated carbon dioxide concentrations. Nature Climate Change, 1(3), 165–169. 10.1038/nclimate1122

[gcb14757-bib-0015] Graham, N. A. J. , Cinner, J. E. , Norström, A. V. , & Nyström, M. (2014). Coral reefs as novel ecosystems: Embracing new futures. Current Opinion in Environmental Sustainability, 7, 9–14. 10.1016/j.cosust.2013.11.023

[gcb14757-bib-0016] Harborne, A. R. , & Mumby, P. J. (2011). Novel ecosystems: Altering fish assemblages in warming waters. Current Biology, 21(19), 9–14. 10.1016/j.cub.2011.08.043 21996508

[gcb14757-bib-0017] Heery, E. C. , Bishop, M. J. , Critchley, L. P. , Bugnot, A. B. , Airoldi, L. , Mayer‐Pinto, M. , … Dafforn, K. A. (2017). Identifying the consequences of ocean sprawl for sedimentary habitats. Journal of Experimental Marine Biology and Ecology, 492, 31–48. 10.1016/j.jembe.2017.01.020

[gcb14757-bib-0018] Heink, U. , & Jax, K. (2019). Going upstream — How the purpose of a conceptual framework for ecosystem services determines its structure. Ecological Economics, 156, 264–271. 10.1016/j.ecolecon.2018.10.009

[gcb14757-bib-0019] Higgs, E. (2017). Novel and designed ecosystems. Restoration Ecology, 25(1), 8–13. 10.1111/rec.12410

[gcb14757-bib-0020] Hobbs, R. J. (2016). Degraded or just different? Perceptions and value judgements in restoration decisions. Restoration Ecology, 24(2), 153–158. 10.1111/rec.12336

[gcb14757-bib-0021] Hobbs, R. J. , Arico, S. , Aronson, J. , Baron, J. S. , Bridgewater, P. , Cramer, V. A. , … Zobel, M. (2006). Novel ecosystems: Theoretical and management aspects of the new ecological world order. Global Ecology and Biogeography, 15(1), 1–7. 10.1111/j.1466-822X.2006.00212.x

[gcb14757-bib-0022] Hobbs, R. J. , Higgs, E. S. , & Hall, C. M. (2013). Defining novel ecosystems In Hobbs R. J., Higgs E. S., & Hall C. M. (Eds.), Novel ecosystems: Intervening in the new ecological world order (pp. 58–60). Hoboken, NJ: Wiley‐Blackwell.

[gcb14757-bib-0023] Hobbs, R. J. , Higgs, E. S. , & Hall, C. M. (2017). Expanding the portfolio: Conserving nature's masterpieces in a changing world. BioScience, 67(6), 568–575. 10.1093/biosci/bix043

[gcb14757-bib-0024] Hobbs, R. J. , Higgs, E. , Hall, C. M. , Bridgewater, P. , Chapin, F. S. , Ellis, E. C. , … Yung, L. (2014). Managing the whole landscape: Historical, hybrid, and novel ecosystems. Frontiers in Ecology and the Environment, 12(10), 557–564. 10.1890/130300

[gcb14757-bib-0025] Hobbs, R. J. , & Kristjanson, L. J. (2003). Triage: How do we prioritize health care for landscapes? Ecological Management & Restoration, 4, S39–S45. 10.1046/j.1442-8903.4.s.5.x

[gcb14757-bib-0026] Holling, C. S. (1973). Resilience and stability of ecological systems. Annual Review of Ecology and Systematics, 4(1), 1–23. 10.1146/annurev.es.04.110173.000245

[gcb14757-bib-0027] Hughes, T. P. , Kerry, J. T. , Baird, A. H. , Connolly, S. R. , Dietzel, A. , Eakin, C. M. , … Torda, G. (2018). Global warming transforms coral reef assemblages. Nature, 556, 492–496. 10.1038/s41586-018-0041-2 29670282

[gcb14757-bib-0028] Jachowski, D. S. , & Kesler, D. C. (2009). Allowing extinction: Should we let species go? Trends in Ecology & Evolution, 24(4), 180 10.1016/j.tree.2008.11.006 19233507

[gcb14757-bib-0029] Jones, G. P. , & Andrew, N. L. (1992). Temperate reefs and the scope of seascape ecology In Battershill C. N., Schiel D. R., Jones G. P., Creese R. G., & MacDiarmid A. B. (Eds.), Proceedings of the second international temperate reef symposium (pp. 63–76). Auckland, NZ: NIWA Marine.

[gcb14757-bib-0030] Joseph, L. N. , Maloney, R. F. , & Possingham, H. P. (2009). Optimal allocation of resources among threatened species: A project prioritization protocol. Conservation Biology, 23(2), 328–338. 10.1111/j.1523-1739.2008.01124.x 19183202

[gcb14757-bib-0031] Keeney, R. L. (2006). Eliciting knowledge about values for public policy decisions. International Journal of Information Technology & Decision Making, 5(4), 739–749. 10.1142/s0219622006002295

[gcb14757-bib-0032] Kenter, J. O. , O'Brien, L. , Hockley, N. , Ravenscroft, N. , Fazey, I. , Irvine, K. N. , … Williams, S. (2015). What are shared and social values of ecosystems? Ecological Economics, 111, 86–99. 10.1016/j.ecolecon.2015.01.006

[gcb14757-bib-0033] Langhamer, O. , Wilhelmsson, D. , & Engström, J. (2009). Artificial reef effect and fouling impacts on offshore wave power foundations and buoys – A pilot study. Estuarine, Coastal and Shelf Science, 82(3), 426–432. 10.1016/j.ecss.2009.02.009

[gcb14757-bib-0034] Marshall, P. A. , & Baird, A. H. (2000). Bleaching of corals on the Great Barrier Reef: Differential susceptibilities among taxa. Coral Reefs, 19(2), 155–163. 10.1007/s003380000086

[gcb14757-bib-0035] Miller, J. R. , & Bestelmeyer, B. T. (2016). What's wrong with novel ecosystems, really? Restoration Ecology, 24(5), 577–582. 10.1111/rec.12378

[gcb14757-bib-0036] Murcia, C. , Aronson, J. , Kattan, G. H. , Moreno‐Mateos, D. , Dixon, K. , & Simberloff, D. (2014). A critique of the ‘novel ecosystem’ concept. Trends in Ecology & Evolution, 29(10), 548–553. 10.1016/j.tree.2014.07.006 25088744

[gcb14757-bib-0037] NERA . (2016). Oil & gas industry competitiveness assessment. Report on the framework, baseline score, insights and opportunities. Kensington, WA: Australian Resources Research Centre.

[gcb14757-bib-0038] Orr, J. C. , Fabry, V. J. , Aumont, O. , Bopp, L. , Doney, S. C. , Feely, R. A. , … Yool, A. (2005). Anthropogenic ocean acidification over the twenty‐first century and its impact on calcifying organisms. Nature, 437(7059), 681–686. 10.1038/nature04095 16193043

[gcb14757-bib-0039] Parr, M. J. , Bennun, L. , Boucher, T. , Brooks, T. , Chutas, C. A. , Dinerstein, E. , … Molur, S. (2009). Why we should aim for zero extinction. Trends in Ecology & Evolution, 24(4), 181 10.1016/j.tree.2009.01.001 19249115

[gcb14757-bib-0040] Pauly, D. (1995). Anecdotes and the shifting baseline syndrome of fisheries. Trends in Ecology & Evolution, 10(10), 430 10.1016/s0169-5347(00)89171-5 21237093

[gcb14757-bib-0041] Perring, M. P. , & Ellis, E. C. (2013). The extent of novel ecosystems: Long in time and broad in space In Hobbs J. R., Higgs E. S., & Hall C. (Eds.), Novel ecosystems: Intervening in the new ecological world order (pp. 66–80). Hoboken, NJ: Wiley‐Blackwell.

[gcb14757-bib-0042] Rappaport, D. I. , Tambosi, L. R. , & Metzger, J. P. (2015). A landscape triage approach: Combining spatial and temporal dynamics to prioritize restoration and conservation. Journal of Applied Ecology, 52, 590–601. 10.1111/1365-2664.12405

[gcb14757-bib-0043] Seastedt, T. R. , Hobbs, R. J. , & Suding, K. N. (2008). Management of novel ecosystems: Are novel approaches required? Frontiers in Ecology and the Environment, 6(10), 547–553. 10.1890/070046

[gcb14757-bib-0044] Shields, D. (2002). The role of values and objectives in communicating indicators of sustainability. Ecological Indicators, 2(1–2), 149–160. 10.1016/s1470-160x(02)00042-0

[gcb14757-bib-0045] Steffen, W. , Broadgate, W. , Deutsch, L. , Gaffney, O. , & Ludwig, C. (2015). The trajectory of the Anthropocene: The great acceleration. The Anthropocene Review, 2(1), 81–98. 10.1177/2053019614564785

[gcb14757-bib-0046] Steffen, W. , Grinevald, J. , Crutzen, P. , & McNeill, J. (2011). The Anthropocene: Conceptual and historical perspectives. Philosophical Transactions of the Royal Society A: Mathematical, Physical and Engineering Sciences, 369(1938), 842–867. 10.1098/rsta.2010.0327 21282150

[gcb14757-bib-0047] Stewart, G. , Mengersen, K. , Mace, G. M. , McNeely, J. A. , Pitchforth, J. , & Collen, B. (2013). To fund or not to fund: Using Bayesian networks to make decisions about conserving our World's endangered species. CHANCE, 26(3), 10–17. 10.1080/09332480.2013.844992

[gcb14757-bib-0048] van der Stap, T. , Coolen, J. W. , & Lindeboom, H. J. (2016). Marine fouling assemblages on offshore gas platforms in the Southern North Sea: Effects of depth and distance from shore on biodiversity. PLoS ONE, 11(1), e0146324 10.1371/journal.pone.0146324 26745870 PMC4706432

[gcb14757-bib-0049] Venton, D. (2013). Forest management plans in a tangle. Nature, 501(7465), 15–16. 10.1038/501015a 24005395

[gcb14757-bib-0050] Wallace, K. J. , & Jago, M. (2017). Category mistakes: A barrier to effective environmental management. Journal of Environmental Management, 199, 13–20. 10.1016/j.jenvman.2017.05.029 28525807

[gcb14757-bib-0051] Wallace, K. J. , Kiatkoski, M. , Rogers, A. , & Jago, M. (2018). Classifying values for planning the conservation and use of natural resources. Working paper 1808. Crawley, Australia: UWA Agricultural and Resource Economics.

[gcb14757-bib-0052] Wallace, K. J. , Wagner, C. , & Smith, M. J. (2016). Eliciting human values for conservation planning and decisions: A global issue. Journal of Environmental Management, 170, 160–168. 10.1016/j.jenvman.2015.12.036 26826807

[gcb14757-bib-0053] Wilhelmsson, D. , Malm, T. , Thompson, R. , Tchou, J. , Sarantakos, G. , McCormick, N. , … Dubi, A. (2010). Greening Blue Energy: Identifying and managing the biodiversity risks and opportunities of offshore renewable energy. Gland, Switzerland: IUCN.

[gcb14757-bib-0054] Wilson, K. A. , & Law, E. A. (2016). Ethics of conservation triage. Frontiers in Ecology and Evolution, 4, 1–8. 10.3389/fevo.2016.00112

